# A quantitative method to the analysis of MLC leaf position and speed based on EPID and EBT3 film for dynamic IMRT treatment with different types of MLC

**DOI:** 10.1002/acm2.12102

**Published:** 2017-05-18

**Authors:** Yinghui Li, Lixin Chen, Jinhan Zhu, Bin Wang, Xiaowei Liu

**Affiliations:** ^1^ School of Physics Sun Yat‐sen University Guangzhou Guangdong China; ^2^ State Key Laboratory of Oncology in South China Sun Yat‐sen University Cancer Center Guangzhou Guangdong China

**Keywords:** EPID, FILM, MLC position, MLC speed

## Abstract

A quantitative method based on the electronic portal imaging system (EPID) and film was developed for MLC position and speed testing; this method was used for three MLC types (Millennium, MLCi, and Agility MLC). To determine the leaf position, a picket fence designed by the dynamic (DMLC) model was used. The full‐width half‐maximum (FWHM) values of each gap measured by EPID and EBT3 were converted to the gap width using the FWHM versus nominal gap width relationship. The algorithm developed for the picket fence analysis was able to quantify the gap width, the distance between gaps, and each individual leaf position. To determine the leaf speed, a 0.5 × 20 cm^2^ MLC‐defined sliding gap was applied across a 14 × 20 cm^2^ symmetry field. The linacs ran at a fixed‐dose rate. The use of different monitor units (MUs) for this test led to different leaf speeds. The effect of leaf transmission was considered in a speed accuracy analysis. The difference between the EPID and film results for the MLC position is less than 0.1 mm. For the three MLC types, twice the standard deviation (2 SD) is provided; 0.2, 0.4, and 0.4 mm for gap widths of three MLC types, and 0.1, 0.2, and 0.2 mm for distances between gaps. The individual leaf positions deviate from the preset positions within 0.1 mm. The variations in the speed profiles for the EPID and EBT3 results are consistent, but the EPID results are slightly better than the film results. Different speeds were measured for each MLC type. For all three MLC types, speed errors increase with increasing speed. The analysis speeds deviate from the preset speeds within approximately 0.01 cm s^−1^. This quantitative analysis of MLC position and speed provides an intuitive evaluation for MLC quality assurance (QA).

## 1 INTRODUCTION

DMLC technology has been widely used in intensity modulated radiation therapy (IMRT) and volumetric modulated arc therapy (VMAT), given its better tumor dose conformity and reduced radiation to the organs at risk.^1^, ^2^ In order to gain the actual clinical advantage from treatment, it must be ensured that the DMLC technology is performed accurately according to the treatment planning parameters.

The precision of DMLC technology depends on the accuracy of the leaf position and speed.^3^ Errors in the leaf position include random (leaf by leaf) and system (entire leaf) errors. The dosimetric effects of random and systematic position errors in dynamic IMRT, have been reported in many studies. Parsai et al.^4^ found that for Elekta MLCi leaves (Elekta, Crawley, UK), a 1‐mm random error in leaf positions could lead to 5% errors in prescribed dose and a 0.5‐mm systematic error in leaf position could result in significant dosimetric deviations; Rangel^5^ found that for Varian Millennium 120‐leaf MLC (Varian, Palo Alto, CA, USA), a 2‐mm random error resulted in negligible changes for all structures of interest and a 0.3‐mm systematic error can lead to 2% errors in equivalent uniform doses (EUDs) of the clinical target. Errors in leaf speed can result in increased beam holds or gap width errors.^6^ The dosimetric effects of leaf speed errors are reported by Daniel et al.^7^ and Huang et al.^8^ An acceptance quantitative criterion has been proposed for MLC leaf position and speed in AAPM Task Group report 142.^6^ Thus, it is necessary to make a quantitative assessment for leaf position and speed in routine MLC QA.

The picket fence is the most commonly used test for the MLC leaf position. Chui et al.^9^ were the first to design a picket fence with DMLC, and the leaf position errors were evaluated by visual inspection. Chang et al.^10^ designed a picket fence with DMLC, and images were acquired by the electronic portal imaging system (EPID) and film for digital analysis. The full‐width half‐maximum (FWHM) values of the gaps and the distance between gaps (interpeak distance) were used to evaluate the consistency of the leaf position, and the standard deviation was used as an evaluation criterion. Given the difference between the FWHM values of the gap and gap width, the system leaf position errors cannot be directly analyzed in a quantitative manner using the FWHM.

A sliding‐window field can be used to determine the MLC leaf speed. Ling et al.^11^ evaluated the MLC leaf speed control during RapidArc. Their field contained multiple DMLC subfields. The exposure dose for each subfield was the same, and the dose rates for each subfield were differenced to combine different leaf speeds. Open‐field profiles of the same size were used as evaluation criteria to determine the dose deviation of the sliding field caused by leaf speed errors.

At present, several MLC types used for clinical applications are primarily provided by two venders: Varian and Elekta. Three MLC types are commonly used: 120 Millennium (Varian, Palo Alto, CA, USA), 80 MLCi/MLCi2 (Elekta Oncology Systems, Crawley, UK), and 160 Agility (Elekta Oncology Systems, Crawley, UK). Therefore, evaluating the leaf position and speed for the above three MLC types have representative significance.

Currently, the tools used for MLC position and speed tests are primarily based on EPID and film methods.^12^, ^13^, ^14^, ^15^, ^16^ As a quick and convenient measurement tool, EPID has been widely used in MLC QA work,^17^, ^18^ and the film approach is also widely used in MLC QA work as a high‐resolution tool.

In this study, a single quantitative evaluation method for determining the leaf position and speed was developed for the above three MLC types. The EPID and EBT3 film approaches were used in this QA work to quantify the leaf position and speed errors. In addition, the results obtained from these two tools were compared.

## 2 MATERIALS AND METHODS

### 2.A Linear accelerator and MLC

The linacs used in this study included Trilogy (Varian, Palo Alto, CA, USA) with 120 Millennium leaves, Synergy (Elekta, Crawley, UK) with 80 MLCi leaves and Versa HD (Elekta, Crawley, UK) with 160 Agility leaves. The 6‐MV photon mode was used for all irradiation. Before the measurement, the machine performance for all linaces has been checked according to the AAPM Task Group report 142.^6^

The Millennium MLC consists of two banks of 60 leaves: the central 40 leaves of each bank are 0.5 cm in width (at the isocenter plane) and the outer 20 leaves are 1.0 cm in width. The minimum gap width formed by the Millennium MLC is 0.5 mm, and the maximum leaf speed is 3.0 cm s^−1^. The MLCi MLC consists of two banks of 40 leaves, and the leaf width is 1.0 cm at the isocenter plane. The minimum gap width formed by the MLCi MLC is 5 mm, and the maximum leaf speed is 2.0 cm s^−1^. The Agility MLC consists of two banks of 80 leaves, and the leaf width is 0.5 cm at the isocenter plane. The minimum gap width formed by the Agility MLC is 4 mm, and the maximum leaf speed is 3.5 cm s^−1^.

### 2.B EPID and film

The Trilogy system includes the aS1000 EPID (Varian, Palo Alto, CA, USA) for megavoltage image acquisition. The source to detector distance (SDD) is 140 cm. The sensitive area of aS1000 is 40 × 30 cm^2^, containing 1024 × 768 pixels, and the pixel pitch is 0.392 × 0.392 mm^2^. Image acquisition was performed in integrated mode, and offset correction, gain correction, and pixel correction were performed for each image. The EPID image data were then back‐projected to the isocenter plane for analysis. A linear relationship between the EPID pixel value and dose value was verified in previous studies of our group.^19^, ^20^ Therefore, to simplify the procedure, pixel values were used to analyze the leaf position and speed. The alignment, SDD, and isocenter position of aS1000 were calibrated before the experimental measurement.

The Synergy and Versa HD systems have the iView GT EPID (Elekta, Crawley, UK) for megavoltage image acquisition. The SDD is 160 cm. The sensitive area of iView GT is 40 × 40 cm^2^, containing 1024 × 1024 pixels, and the pixel pitch is 0.4 × 0.4 mm^2^. Image acquisition was performed in integrated mode, and offset correction, gain correction, and pixel correction were performed for each image. The EPID image data were then back‐projected to the isocenter plane for analysis. A linear relationship between the EPID pixel value and dose value was verified in previous studies of our group.^19^, ^20^ Therefore, to simplify the procedure, the pixel values were used to analyze the leaf position and speed. The alignment, SDD, and isocenter position of iView GT were calibrated before the experimental measurement.

GAFCHROMIC EBT3 (Ashland ISP Advanced Materials) film with sheet dimensions of 20.32 × 25.40 cm^2^ was used for the acquisition of megavoltage images for all linacs. The films were irradiated at SDD = 100 cm with a 0.5‐cm solid water phantom (PTW Freiburg, Freiburg, Germany) buildup for the leaf position test and with a 1.5‐cm buildup for leaf speed test. The scan resolution of the film was 72 dpi, and the pixel pitch was 0.353 × 0.353 mm^2^. FILMQA PRO software (Ashland Inc., Wayne, NJ, USA) was used for dose conversion, and the dose maps were exported to MATLAB in the comma‐separated value (CSV) format for leaf position and speed analysis.

### 2.C Designs of MLC position and speed test

#### 2.C.1 MLC position test

In this work, the field size was 14 × 24 cm^2^ and the gantry was set at zero degrees. The collimators of Synergy and Versa HD systems were set at zero degrees, and the Trilogy system was set at 90° to include as many MLC leaves as possible. Eight pickets were formed by a sliding gap stopping at every 2 cm. The total beam weight is 1 (total monitor units (Mus) = 1000 MU). The weight for each picket is 0.09 (0.09 × 8 = 0.72), and the weight for each 2‐cm sliding distance is 0.04 (0.04 × 7 = 0.28).

Several picket fence fields have been designed for calibrating the FWHM versus the nominal gap width, and each picket fence field contains only a nominal gap width. The nominal gap widths for the three MLC types are 1–10 mm (Millennium), 6–12 mm (MLCi), and 4–12 mm (Agility). All picket fence fields for calibration should be conducted immediately after the MLC has been calibrated. The machine QA tests before establishing the gap width calibration function were based on Mubata^21^ for Varian system and Autocal^22^ for Elekta system.

A picket fence field with a nominal gap width of 5 mm was chosen as the test field for the Millennium MLC position, and a picket fence field with a nominal gap width of 10 mm was chosen as the testing field for the MLCi and Agility MLC position.

**Figure 1:**
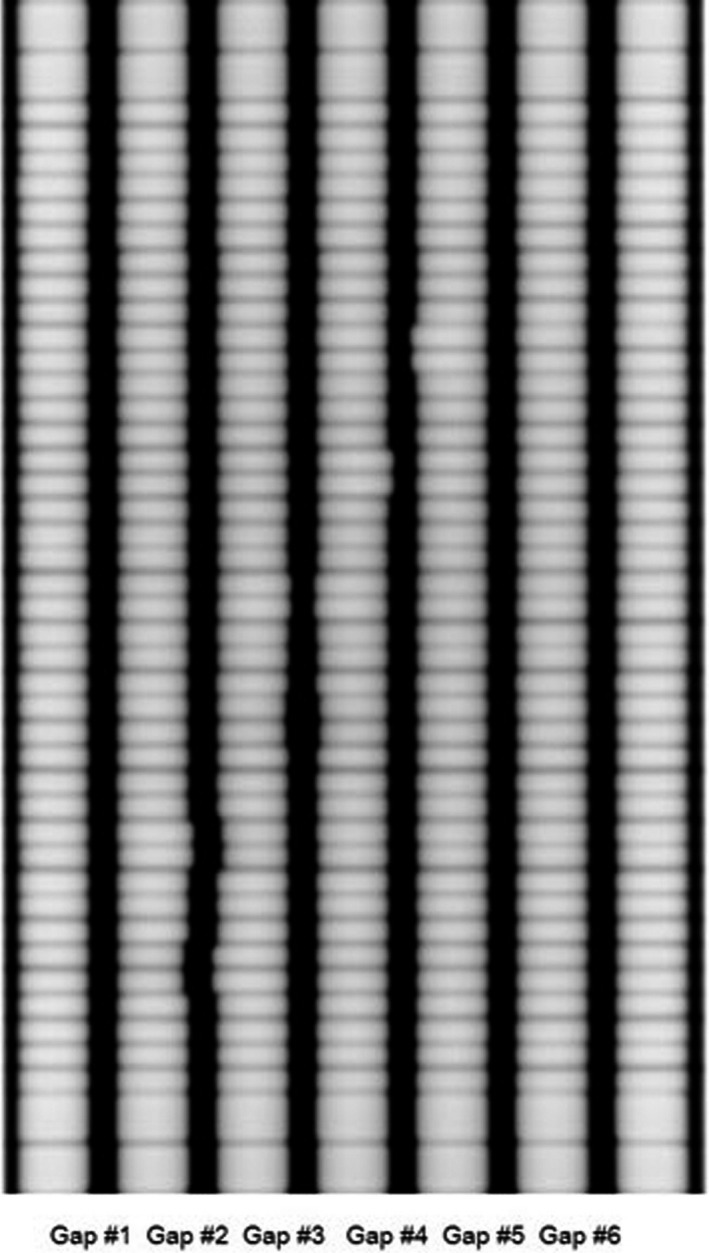
Image of the picket fence test for the Millennium DMLC position QA. Gap 2, a deviation of ± 1 mm for the distance between gaps; gap 3, a deviation of ± 1 mm for the gap width; gap 4, a deviation of 1 mm position for only the left or right leaf.

To verify the accuracy of the MLC position analysis presented in this work, a deviation of ± 1 mm for the distance between the gaps, of ± 1 mm for the gap width, and of 1 mm for the position for only left or right leaves were introduced in gap 2, gap 3, and gap 4, respectively, as shown in Fig. 1, using the EPID image of the Millennium MLC as an example. Each measurement was repeated six times to reduce any uncertainty.

#### 2.C.2 MLC speed test

In this work, a 0.5 × 20 cm^2^ MLC‐defined gap was moved across a 14 × 20 cm^2^ symmetric field at a constant speed. The linacs were run at a fixed‐dose rate (600 MU/min theoretical dose rate), and both the collimators and gantry were set at zero degrees.

The use of different monitor units for this test led to different leaf speeds. The dose rate of the Trilogy was 600 MU/min, and the monitor units were set to 47, 70, 140, and 280 MU, with nominal leaf speeds of approximately 2.98, 2.00, 1.00, and 0.50 cm s^−1^. The dose rate of the Synergy system was 621 MU/min, and the monitor units were set to 76, 96, 145, and 290 MU. The nominal leaf speeds were approximately 1.91, 1.51, 1.00, and 0.50 cm s^−1^. The dose rate of the Versa HD was 578 MU/min, and the monitor units were set to 39, 54, 135, and 270 MU. The nominal leaf speeds were approximately 3.46, 2.50, 1.00, and 0.50 cm s^−1^.

We then sought to verify the accuracy of the MLC speed analysis in this work. For a sliding field with a normal speed of 1 cm s^−1^, the monitor unit deviations (3, 2, 1, −1, −2, and −3 MU) were introduced into the 3‐cm length of the center of the sliding field. The nominal speeds were approximately 0.91, 0.94, 0.97, 1.03, 1.07, and 1.11 cm s^−1^. The profiles are presented in Fig. 6.

### 2.D Data analysis

To reduce the effect of interleaf leakage,^10^ data regarding the leaf center position from each leaf pair were used for analysis. In addition, to reduce the effect of noise,^23^ the middle nine profiles were averaged in the direction perpendicular to the leaf motion, and the average values were used for analysis.

#### 2.D.1 MLC position test

The six gaps (gaps 1—6 in Fig. 1) in the picket fence were used as analysis data for the MLC position. A QA of the MLC positions was performed based on the results for the distance between the gaps and the gap width. If leaf position errors were found, determining the individual leaf position for each leaf pair was necessary.

##### Determinations of the FWHM and the distance between gaps

For each leaf pair, the maximum and minimum values for each gap were determined through a search. The average maximum and minimum values were defined as the 50% maximum value. The positions of the 50% maximum value on both sides of each gap were determined by linear interpolation, and the FWHM values were then determined. The mean position of the right and left 50% maximum of each gap was defined as the peak position. The distance between the adjacent peaks was defined as the distance between the gaps.

##### Calibration of gap width

**Figure 2:**
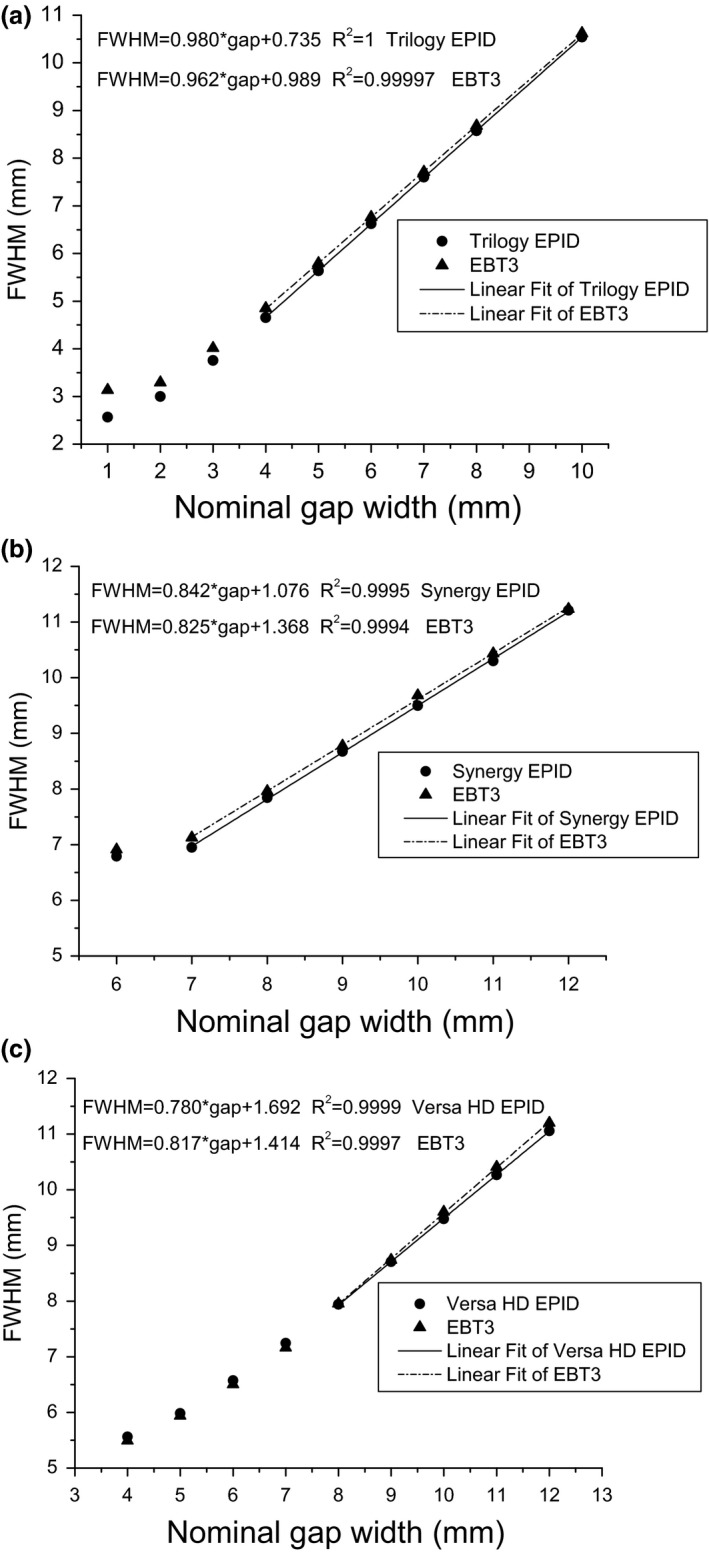
The *FWHM* ‐ *ngap*_*w*_ relationship for the three MLC types measured by the EPID and EBT3 film methods. (a) Millennium MLC, (b) MLCi MLC, and (c) Agility MLC.

The picket fence images from Trilogy, Synergy, and Versa HD each contained a 264 (44 leaf pairs × 6 pickets), 144 (24 leaf pairs × 6 pickets), and 288 (48 leaf pairs × 6 pickets) FWHM values, respectively. The average FWHM over all leaf pairs was used to calibrate the gap width. According to the FWHM versus nominal gap width relationship (Fig. 2), an appropriate calibration function ($ngap_{w}=f\left(FWHM\right)$,$ngap_{w}$ is the nominal gap width) was selected. The measurement gap width ($mgap_{w}$) for the position test field can be calculated from the calibration function.

##### Determination of individual leaf position

The position errors can be easily detected by measuring the gap width and distance between gaps for each leaf pair. The next study will determine the position for each individual leaf in the testing field. Using one leaf pair as an example, the individual leaf position on the left and right of each gap can determined by formulas (1) and (2).

(1) $$P_{L}=P-\frac{mgap_{w}}{2}$$

(2) $$P_{R}=P+\frac{mgap_{w}}{2},$$

where $P_{L}$ and $P_{R}$ are the leaf positions on the left and right of the gap, respectively, and P is the peak position of the gap.

#### 2.D.2 MLC speed test

One leaf pair was taken as an example to analyze the leaf speed. For a fixed‐dose rate, the dose contribution at the j‐th position was determined as follows:

(3) $$D_{j}=\Delta t_{j}\cdot \overset{∙}{D_{j}}+(t_{total}-\Delta t_{j})\cdot \overset{∙}{D_{{}_{j}}^{tran}},$$

where $\overset{∙}{D_{j}}$ is the irradiation dose rate at the j‐th position; $\overset{∙}{D_{{}_{j}}^{tran}}=T_{MLC}\cdot \overset{∙}{D_{j}}$ is the transmission dose rate at the j‐th position; $T_{MLC}$ is the MLC transmission evaluated by measuring the ratio of the MLC closed field and open field; $t_{total}$ is the total irradiation time of the sliding field; $\Delta t_{j}=\frac{\Delta S}{V_{j}}$, where ΔS is the width of the sliding gap, set at 5 mm in this work; $V_{j}$ is the average speed of the MLC‐defined gap moving across position j‐th; and $\Delta t_{j}$ is the irradiation time of the MLC‐defined gap moving across position j‐th.

The dose rate ratio of the j‐th position to the center position of the profile was defined as $w_{{}_{j}}=\frac{\overset{∙}{D_{j}}}{\overset{∙}{D_{0}}}$, where $\overset{∙}{D_{0}}$ is the irradiation dose rate at the center position of the profile. If there are no leaf speed errors, the normalized sliding profile should be the same as the profile of the corresponding open MLC field (Ling et al. 2008). Therefore, the profile of the corresponding open MLC field was introduced as a comparison standard, and the $w_{{}_{j}}$ values of the sliding profile were determined by the dose ratio of the corresponding open MLC field at the same position: $w_{{}_{j}}=\frac{\overset{∙}{D_{j}}}{\overset{∙}{D_{0}}}=\frac{D_{j}^{open}}{D_{0}^{open}}$. Thus, the leaf speed at the j‐th position can be defined as

(4) $$V_{j}=\frac{K_{{}_{0}}}{H_{{}_{j}}},$$

where $H_{{}_{j}}=\left(\frac{D_{j}}{w_{{}_{j}}}-t_{total}\cdot T_{MLC}\cdot \overset{∙}{D_{{}_{0}}}\right)$ is inversely proportional to the speed $V_{j}$; $t_{total}\cdot T_{MLC}\cdot \overset{∙}{D_{{}_{0}}}$ is the transmission dose; and $K_{0}=\Delta S\cdot \left(1-T_{MLC}\right)\cdot \overset{∙}{D_{0}}$ can be regarded as a constant when the dose rate of the linear accelerator is fixed. The $K_{{}_{0}}$value can be determined by calibrating the nominal leaf speed versus the $H_{{}_{0}}$ value.

## 3 RESULTS

### 3.A MLC position test

#### 3.A.1 Determination of the gap width calibration function

The FWHM values of the corresponding $ngap_{w}$ measured by the EPID and EBT3 film methods are presented in Tables 1, 2, 3, providing the measured FWHM values for the Millennium MLC, the MLCi MLC, and the Agility MLC, respectively. The 2 SD values are also provided in Tables 1, 2, 3. The FWHM versus $ngap_{w}$relationship is presented in Fig. 2. Figures 2(a)–2(c) correspond to Tables 1, 2, 3, respectively. Figure 2 shows that the $FWHM-ngap_{w}$ relationships among the three MLC types changed from nonlinear to linear with increasing $ngap_{w}$ values. The nonlinear–linear turning point for the EPID and EBT3 results were the same for each MLC type: an $ngap_{w}$ value of 4 mm for Millennium, 7 mm for MLCi, and 8 mm for Agility. The gap width of the MLC test field was selected in the linear range, and a simple linear function was used as the calibration function: $FWHM=a\cdot ngap_{w}+b$, where a and b were the fitting coefficients.

**Table 1:** The FWHM of the corresponding *ngap*_*w*_ of the Millennium MLC measured by the EPID and EBT3 film methods, reported as the mean ± 2 SD

*ngap*_*w*_	FWHM (EPID)	FWHM (EBT3)
1.00 mm	2.57 ± 0.08 mm	3.14 ± 0.25 mm
2.00 mm	3.00 ± 0.13 mm	3.29 ± 0.14 mm
3.00 mm	3.76 ± 0.14 mm	4.02 ± 0.14 mm
4.00 mm	4.66 ± 0.16 mm	4.85 ± 0.16 mm
5.00 mm	5.64 ± 0.17 mm	5.79 ± 0.17 mm
6.00 mm	6.62 ± 0.17 mm	6.76 ± 0.18 mm
7.00 mm	7.60 ± 0.16 mm	7.71 ± 0.19 mm
8.00 mm	8.58 ± 0.16 mm	8.68 ± 0.17 mm
10.00 mm	10.54 ± 0.17 mm	10.62 ± 0.17 mm

**Table 2:** The FWHM of the corresponding *ngap*_*w*_ of the MLCi MLC measured by the EPID and EBT3 film methods, given as the mean ± 2 SD

*ngap*_*w*_	FWHM (EPID)	FWHM (EBT3)
6.00 mm	6.79 ± 0.15 mm	6.91 ± 0.28 mm
7.00 mm	6.95 ± 0.19 mm	7.13 ± 0.19 mm
8.00 mm	7.84 ± 0.16 mm	7.96 ± 0.20 mm
9.00 mm	8.68 ± 0.18 mm	8.78 ± 0.21 mm
10.00 mm	9.50 ± 0.18 mm	9.68 ± 0.19 mm
11.00 mm	10.30 ± 0.17 mm	10.44 ± 0.21 mm
12.00 mm	11.21 ± 0.20 mm	11.23 ± 0.16 mm

**Table 3:** The FWHM of the corresponding *ngap*_*w*_ of the Agility MLC measured by the EPID and EBT3 film methods, given as the mean ± 2 SD

*ngap*_*w*_	FWHM (EPID)	FWHM (EBT3)
4.00 mm	5.56 ± 0.16 mm	5.49 ± 0.18 mm
5.00 mm	5.98 ± 0.17 mm	5.93 ± 0.19 mm
6.00 mm	6.57 ± 0.18 mm	6.50 ± 0.22 mm
7.00 mm	7.25 ± 0.21 mm	7.16 ± 0.24 mm
8.00 mm	7.94 ± 0.21 mm	7.96 ± 0.24 mm
9.00 mm	8.71 ± 0.21 mm	8.74 ± 0.21 mm
10.00 mm	9.48 ± 0.22 mm	9.60 ± 0.25 mm
11.00 mm	10.27 ± 0.22 mm	10.41 ± 0.27 mm
12.00 mm	11.06 ± 0.21 mm	11.20 ± 0.24 mm

#### 3.A.2 Statistical analysis of the MLC position

Table 4 presents the leaf position results for the three MLC types measured by the EPID and EBT3 film methods, including the gap width and the distance between gaps. The average data for all leaf pairs ± 2 SD are provided. The measurement results from both EPID and EBT3 film in this work are consistent with the preset values; the differences were less than 0.1 mm. The ± 2 SD values are provided to illustrate the consistency of the MLC position. The 2 SD results measured by the EPID and EBT3 film methods were similar (less than 0.1 mm). The gap width of the Millennium, MLCi, and Agility MLC meet the 2 SD at 0.2, 0.4, and 0.4 mm, respectively. The distance between gaps meets the 2 SD at 0.1, 0.2, and 0.2 mm, respectively.

**Table 4:** Leaf position results for the three MLC types measured by the EPID and EBT3 film methods, including the gap width and the distance between gaps. The average data over all leaf pairs ± 2 SD are provided

	Millennium		MLCi		Agility	
	EPID	EBT3	EPID	EBT3	EPID	EBT3
Gap width (mm)	5.01 ± 0.19	5.02 ± 0.22	10.01 ± 0.25	10.04 ± 0.28	9.97 ± 0.32	9.98 ± 0.32
Distance between gaps (mm)	20.00 ± 0.06	20.01 ± 0.09	20.05 ± 0.23	20.03 ± 0.18	20.02 ± 0.16	20.01 ± 0.18

The results of the introduced deviations of the picket fence test field for the three MLC types measured by EPID and EBT3 film are presented in Table 5. The average of six measurements is presented with ± 2 SD. The average difference between the nominal and measured values is less than 0.1 mm, indicating that the algorithm developed for the picket fence analysis based on the EPID and film methods can accurately detect the MLC leaf position, including the gap width, the distance between gaps, and each individual leaf position.

**Table 5:** Results of the introduced deviations of the picket fence test field for the three MLC types measured by the EPID and EBT3 film methods, including the gap width, the distance between gaps, and each individual leaf position. The average of six measurements is presented with ± 2 SD

	Deviation of gap width		Deviation of distance between gaps		Deviation of individual leaf position	
	−1 mm	+1 mm	−1 mm	+1 mm	1 mm	0 mm
Millennium						
EPID	−0.97 ± 0.03	1.03 ± 0.03	−0.95 ± 0.03	1.02 ± 0.02	1.01 ± 0.02	0.03 ± 0.02
Film	−0.96 ± 0.09	1.09 ± 0.12	−0.95 ± 0.05	0.96 ± 0.05	0.98 ± 0.05	0.08 ± 0.05
MLCi						
EPID	−1.01 ± 0.07	1.06 ± 0.06	−1.09 ± 0.11	0.92 ± 0.08	1.01 ± 0.10	0.06 ± 0.02
Film	−0.99 ± 0.12	0.99 ± 0.15	−0.96 ± 0.13	0.95 ± 0.11	0.91 ± 0.18	0.08 ± 0.04
Agility						
EPID	−0.97 ± 0.06	0.98 ± 0.05	−1.06 ± 0.07	0.95 ± 0.05	1.08 ± 0.03	0.06 ± 0.03
Film	−0.99 ± 0.12	1.01 ± 0.09	−0.97 ± 0.08	0.98 ± 0.10	1.05 ± 0.04	0.03 ± 0.04

### 3.B Speed test

Table 6 presents the transmission (not including the interleaf transmission) for the three MLC types measured by the EPID and EBT3 film methods. The transmissions in Table 6 were used to determine $H_{{}_{j}}$ in eq. (4). Given the difference in measurement conditions (EPID with no additional buildup and EBT3 with 1.5‐cm buildup), the transmissions measured by EPID and EBT3 film were also different. The transmission of the Agility MLC measured by EPID is negligible.

**Table 6:** Transmission for the three MLC types measured by the EPID and EBT3 film methods, not including the interleaf transmission. EPID with no additional buildup and EBT3 with 1.5‐cm buildup

	Millennium	MLCi	Agility
EPID	1.11%	0.43%	0.02%
Film	1.50%	0.97%	0.53%

**Figure 3:**
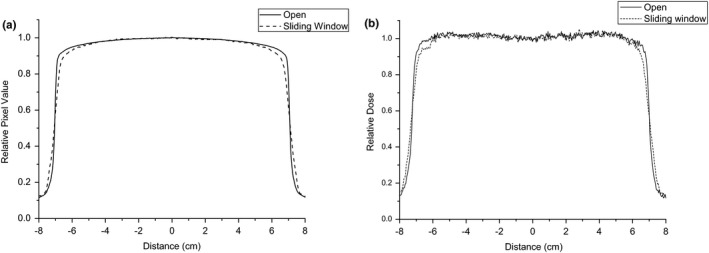
Verification of the consistency between the sliding and open‐field profiles measured by EPID and EBT3 film. (a) EPID, (b) film.

A verification of the consistency between the sliding and open‐field profile measured by EPID and EBT3 film is presented in Fig. 3. Within the central 80% of the field width in the direction of leaf motion, the two profiles matched closely. There was a difference between the sliding and open field on the profile shoulders, primarily because the irradiation times for the beginning and end positions were less than the time in which the MLC moved a distance of ΔS. Therefore, the open‐field profile can be used as a standard to determine the dose‐rate ratio $w_{{}_{j}}$, and the measurement data ranging from −5 to 5 cm were used for leaf speed analysis.

**Figure 4:**
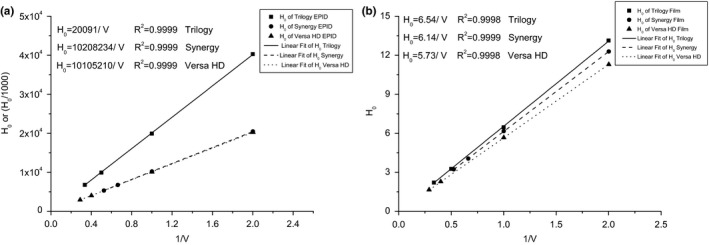
The determination of $K_{{}_{0}}$. The $K_{{}_{0}}$ value was calculated as the slope of the fitting lines. One‐thousandth of the EPID $H_{{}_{0}}$ value for Synergy and Versa HD was used for the vertical coordinates. (a) EPID, (b) EBT3 film.

The $K_{{}_{0}}$ value in eq. (5) was experimentally determined by creating sliding fields with different nominal leaf speeds. Given that the leaf speed is inversely proportional to $H_{{}_{0}}$, we can plot the reciprocal of the nominal speed versus the measured value of $H_{{}_{0}}$. The slopes of the fitting lines are presented in Fig. 4. The coefficient of determination (R‐square) is also provided in Fig. 4. For simplicity, the fitting lines of the three MLC types were plotted in one image, and one‐thousandth of the $H_{{}_{0}}$ value for Synergy and Versa HD, as measured by EPID, was used as the vertical coordinate.

**Figure 5:**
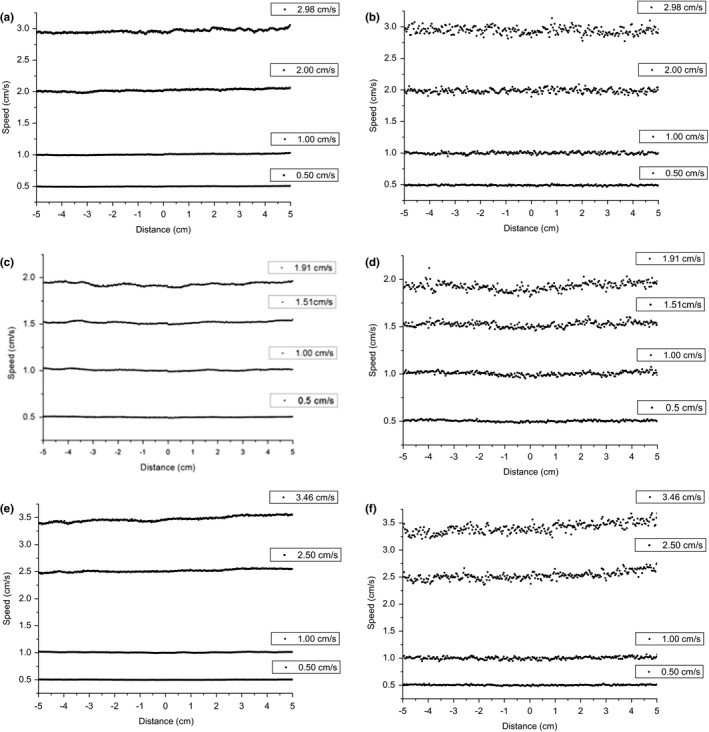
Leaf speed analysis results for the three MLC types measured by EPID and EBT3 film, using one leaf pair as an example. (a) EPID and (b) film for Millennium, (c) EPID and (d) film for MLCi, (e) EPID and (f) film for Agility.

Figures 5(a)–5(f) present the leaf speed analysis results for the three MLC types measured by EPID and EBT3 film, using a leaf pair as an example. The results measured by EPID and EBT3 film are shown in Figs. 5(a) and 5(b) for the Millennium MLC, in Figs. 5(c) and 5(d) for the MLCi MLC, and in Figs. 5(e) and 5(f) for the Agility MLC, respectively. As noted in Fig. 5, along the direction of MLC motion, the variation tendency of the speed profiles from the EPID and EBT3 results is consistent. However, the fluctuation ranges of the EBT3 results are greater than those of the EPID results. Four different speeds were measured for each MLC type. A common feature of the three MLC types is that the fluctuation range of leaf speed increases with increasing leaf speed. Detailed statistical results are presented in Table 7.

**Table 7:** The statistical results of leaf speed for the three MLC types measured by EPID and EBT3 film, using one leaf pair as an example. The averages of the speed profiles are presented with ± 2 SD

	Millennium		MLCi		Agility	
	EPID	EBT3	EPID	EBT3	EPID	EBT3
0.50 cm s^−1^	0.50 ± 0.01	0.49 ± 0.02	0.50 ± 0.01	0.51 ± 0.02	0.50 ± 0.00	0.50 ± 0.02
1.00 cm s^−1^	1.01 ± 0.02	1.00 ± 0.03	1.00 ± 0.02	1.01 ± 0.05	1.01 ± 0.01	1.00 ± 0.05
1.51 cm s^−1^			1.52 ± 0.02	1.53 ± 0.07		
1.91 cm s^−1^			1.93 ± 0.04	1.93 ± 0.11		
2.00 cm s^−−1^	2.02 ± 0.04	1.99 ± 0.07				
2.50 cm s^−1^					2.52 ± 0.05	2.53 ± 0.14
2.98 cm s^−1^	2.97 ± 0.05	2.94 ± 0.12				
3.46 cm s^−1^					3.47 ± 0.10	3.41 ± 0.18

**Figure 6:**
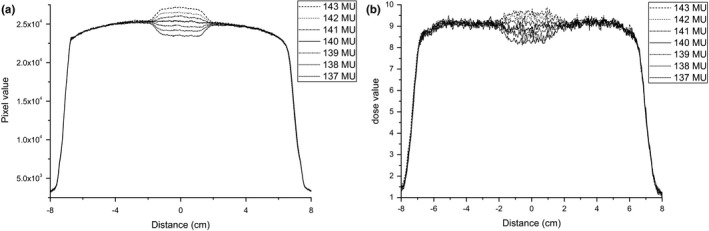
The sliding profiles for Trilogy at the normal leaf speed and introduced leaf speed difference measured by EPID and EBT3 film. (a) EPID, (b) EBT3 film.

The sliding profiles for the Trilogy MLC at the normal leaf speed and introduced leaf speed difference measured by EPID and EBT3 film are presented in Fig. 6. The profiles for Synergy and Versa HD are similar to those for Trilogy. As noted in Fig. 6, both the EPID and EBT3 film can sensitively detect dose changes. Compared with the theoretical speed ratios (0.91, 0.94, 0.97, 1.03, 1.07, 1.11) of the introduced difference to the normal speed, the corresponding dose ratios (normal to introduced difference) for Trilogy measured by EPID are 0.94, 0.96, 0.98, 1.02, 1.05, and 1.08, respectively. The dose ratios differ from the theoretical speed ratios, and the difference increases with increasing theoretical speed ratios. The other measurement results are similar to the EPID results for Trilogy except for the EPID results for Versa HD. The corresponding dose ratios for Versa HD measured by EPID are 0.92, 0.95, 0.98, 1.03, 1.07, and 1.10, respectively. The dose ratios are consistent with the theoretical speed ratios, with a difference of 0.01. These results are attributed to the fact that the EPID‐measured transmission of the Agility MLC is very small (0.02%). The transmission dose is approximately 0.5% in the dose for the sliding field, and the influence of transmission is negligible. However, for the other measurement, the transmission dose is greater than 10%, and an accurate speed analysis must consider the influence of transmission. Detailed statistical results are presented in Table 8.

**Table 8:** The statistical results for the introduced leaf speed difference for the three MLC types measured by EPID and EBT3 film. The average of the introduced difference speed profile is presented with ± 2 SD

	Millennium		MLCi		Agility	
	EPID	EBT3	EPID	EBT3	EPID	EBT3
0.91 cm s^−1^	0.92 ± 0.01	0.91 ± 0.03	0.92 ± 0.01	0.92 ± 0.03	0.92 ± 0.01	0.92 ± 0.03
0.94 cm s^−1^	0.95±0.01	0.94 ± 0.04	0.94 ± 0.01	0.94 ± 0.03	0.95 ± 0.01	0.94 ± 0.04
0.97 cm s^−1^	0.98 ± 0.01	0.97 ± 0.03	0.97 ± 0.01	0.97 ± 0.04	0.98 ± 0.01	0.96 ± 0.04
1.03 cm s^−1^	1.03 ± 0.01	1.03 ± 0.03	1.03 ± 0.02	1.04 ± 0.03	1.03 ± 0.01	1.03 ± 0.04
1.07 cm s^−1^	1.07 ± 0.01	1.06 ± 0.05	1.06 ± 0.01	1.07 ± 0.03	1.07 ± 0.01	1.06 ± 0.04
1.11 cm s^−1^	1.10 ± 0.01	1.10 ± 0.04	1.09 ± 0.02	1.10 ± 0.04	1.10 ± 0.01	1.10 ± 0.04

## 4 DISCUSSION

In this study, it was found that the method can quantify the MLC leaf position and speed accurately by using EPID and EBT3 film. Three MLC types were investigated in this study. It was found that the position and speed QA for all of these MLC types can be achieved using this method.

Compared with the 1‐mm gap width utilized for the picket fence test in many studies,^9^, ^10^, ^18^ the gap widths in this work (5 mm for Varian and 10 mm for Elekta) were significantly greater. The larger gap width in our work can be calibrated by a linear function (as shown in Fig. 2), which can be fitted with fewer data points compared with those for a nonlinear calibration; thus, the calibration is simple and quick. The larger gap width may reduce the ability of visual inspection (in Fig. 1, the 0.5 mm errors can still be clearly observed), but the sensitivity of the data analysis is not affected.

Using the FWHM to calibrate gap width, the advantage is that the FWHM value will not be affected by the variation of the beam dose or the detector sensitivity. For Millennium MLC, the FWHM values between the central 40 leaves (0.5 cm) and the outer 20 leaves (1.0 cm) were not different, the gap width can be calibrated using the same function. After gap width calibration, the quantitative results (mean and standard deviation) for picket fence test can be given. The mean values can reflect the MLC system errors and the standard deviation can reflect the MLC random errors. According to the reports of Parsai et al.^4^ and Rangel^5^ on the clinical dosimetric effects of MLC systematic and random errors, in our test, the systematic errors are required to be less than 0.3 mm and the random errors (2 SD) are required to be less than 1 mm.

From the test results for the EPID and EBT3 film methods, presented in Table 4, the sensitivity of EPID and EBT3 is similar for MLC position QA. However, to improve the signal‐to‐noise ratio of EBT3, 1000 MU was used as the irradiation of the picket fence field in this work. If only the EPID is used, a smaller MU (200 MU) can be employed for MLC position QA.

For the evaluation of leaf speed, the effect of transmission has been considered in this study. If the effect of transmission not considered, the simple visual inspection for leaf speed may not be affected (in Fig. 6), but the quantitative results have significant differences with the introduced speed errors. The difference was increased with increasing speed errors. Therefore, an accurate quantitative analysis of leaf speed must consider the impact of transmission.

Although the analysis results in this work have demonstrated that both EPID and EBT3 film can be used for MLC speed QA, the lower signal‐to‐noise ratio is an issue for leaf speed QA in EBT3 film. To improve the signal‐to‐noise ratio, each film in this study was repeatedly irradiated (more than three times), and the average data were used for analysis. Both the EPID and EBT3 results indicate that the leaf speed errors increase with increasing leaf speed. The same results are evident in the study by Rowshanfarzad et al.^18^

Due to the limitation from size or SDD of EPID and film, the iView GT EPID and EBT3 film need two measurements to evaluate all MLC leafs. The aS1000 EPID can evaluate all MLC leafs in a single measurement with the conditions of 90° collimator angle and SDD100 cm.

## 5 CONCLUSION

This study provides a quantitative analysis method for MLC position and speed QA based on the EPID and EBT3 film approaches. The method can be applied to multiple MLC types and ensures safe and reliable use of the DMLC IMRT.

## ACKNOWLEDGMENTS

This work was supported by the Combination of Guangdong Province and Ministry of Education Research project (2012B091000144), Natural Science Foundation of Guangdong Province (2014A030310188 and 2016A030313276), and Guangzhou Science and Technology project (20160701168).

## CONFLICT OF INTEREST

The authors declare no conflicts of interest.
